# Cost-Aware Scheduling Under Latency Constraints for Multi-View 3D Reconstruction Across the Edge–Cloud Continuum

**DOI:** 10.3390/s26134317

**Published:** 2026-07-07

**Authors:** Ivan Čilić, Ivana Podnar Žarko, Mario Kušek, Josip Štajdohar

**Affiliations:** 1 Faculty of Electrical Engineering and Computing, University of Zagreb, HR-10000 Zagreb, Croatia; ivana.podnar@fer.hr (I.P.Ž.); mario.kusek@fer.hr (M.K.); 2 Delta Reality, HR-10000 Zagreb, Croatia; jstajdoh@deltareality.com

**Keywords:** multi-view 3D reconstruction, cost-aware scheduling, latency constraints, edge computing

## Abstract

Learning-based multi-view 3D reconstruction pipelines, such as transformer-based approaches, enable the accurate reconstruction of 3D scenes from multiple images, but their deployment across the edge–cloud continuum is challenging due to high computational demands and large intermediate data transfers. Effective pipeline scheduling in the continuum must therefore balance latency constraints with the cost of cloud resource usage. In this work, we address cost-aware scheduling under latency constraints for a multi-stage 3D reconstruction pipeline consisting of depth estimation, transformer-based multi-view fusion, and point cloud merging with export to a rendering-ready representation. We implement a service-oriented pipeline where each stage can be executed either on edge or cloud nodes, and we experimentally characterize its performance on representative hardware platforms. The results show a strong imbalance between the computational time and communication latency across platforms, mainly due to large intermediate data. Based on these insights, we propose an online scheduler that dynamically selects stage placements to minimize the cloud cost while satisfying latency constraints. The scheduler incorporates a top-*K* edge selection mechanism that reduces the decision complexity by jointly considering the network conditions and node utilization. Simulation results parameterized with real-system measurements show that the proposed approach effectively reduces cloud usage while meeting latency constraints, outperforming the baseline strategies based on single-node pipeline execution.

## 1. Introduction

Multi-view 3D reconstruction is a fundamental problem in computer vision, enabling the recovery of geometric scene representations from multiple images. It plays a key role in applications such as robotics, autonomous navigation, digital twins, and immersive media such as AR/VR [1,2,3]. Traditional reconstruction pipelines based on Structure-from-Motion (SfM) and multi-view stereo (MVS) achieve high accuracy but are computationally expensive and difficult to deploy in latency-sensitive environments [4]. Recent advances in learning-based methods, particularly those based on deep neural networks and transformers, have significantly improved reconstruction robustness and efficiency, enabling near-real-time performance in controlled settings [5].

In many emerging applications, such as robotics and AR/VR, image data are captured across multiple devices and must be processed under strict latency constraints, which often requires edge processing and efficient resource allocation. In such scenarios, reconstruction pipelines are no longer limited to single-machine execution but instead span a heterogeneous edge–cloud continuum [6], where computation can be performed either on resource-constrained edge nodes or on more powerful cloud infrastructure.

However, distributing multi-stage reconstruction pipelines across heterogeneous resources introduces a significant systems challenge: how to assign each pipeline stage to an execution node such that service-level objectives (SLOs), e.g., latency deadlines, are satisfied while minimizing the use of costly cloud resources, whose strong computational capabilities come at a significantly higher operational cost compared to edge devices. This problem is particularly challenging due to the heterogeneity of compute nodes in the continuum and the non-negligible communication overhead between stages.

In learning-based reconstruction pipelines, these challenges are further increased by differences between individual stages. While some stages are computation-intensive but produce relatively compact outputs, others generate large intermediate representations that are expensive to transmit over the network. For example, depth estimation can produce dense per-pixel outputs that significantly increase the data volume, making naive offloading strategies that ignore communication costs inefficient. Prior work has shown that the communication overhead can dominate end-to-end latency if not carefully managed [7]. Unlike traditional workflow scheduling problems, learning-based multi-view reconstruction pipelines exhibit highly asymmetric communication patterns. In particular, intermediate representations generated by depth estimation and fusion stages may exceed the sizes of the original input images by an order of magnitude, making communication costs an important factor in placement decisions. Consequently, minimizing cloud usage while satisfying latency constraints requires the joint consideration of the computation latency, communication overhead, queuing effects, and pipeline structure.

Existing approaches have addressed aspects of this problem through latency optimization [4] or adaptive resource management [8], but they often focus either on algorithmic improvements or on system-level heuristics in isolation. As a result, there remains a gap in jointly optimizing computation placement, communication costs, and latency constraints for modern learning-based reconstruction pipelines.

In this paper, we address this challenge by proposing a cost-aware scheduling framework for multi-view 3D reconstruction across the edge–cloud continuum under latency constraints. Our approach models both the computation and communication costs of individual pipeline stages and dynamically determines their placement based on current system conditions. The scheduler minimizes cloud resource usage among all feasible placements while satisfying latency constraints, explicitly accounting for queuing effects, network variability, and the structure of the pipeline.

We implement the proposed framework on a modular pipeline composed of depth estimation, transformer-based multi-view fusion, and point cloud merging with export to a 3D Gaussian Splatting representation. Through experimental evaluation on a heterogeneous edge–cloud setup and a simulator parameterized with real measurements, we demonstrate that the proposed approach effectively minimizes cloud usage while meeting latency deadlines. In particular, the proposed scheduler reduces cloud utilization by 50–70%, depending on the workload and topology, outperforming an edge-greedy scheduling strategy.

To the best of our knowledge, no prior work has explicitly addressed cloud cost-aware scheduling under latency SLO constraints for multi-view 3D reconstruction pipelines deployed across the edge–cloud continuum. The main contributions of this work are as follows:

- We formalize the problem of cost-aware scheduling under latency constraints for learning-based multi-view 3D reconstruction pipelines in the edge–cloud continuum.
- We propose a benchmark-aware online scheduling algorithm that exploits the pipeline structure, communication characteristics, and resource availability to reduce the cloud cost while satisfying latency constraints.
- We evaluate the proposed approach on a real edge–cloud system and in a simulation environment parameterized using real-system performance measurements, demonstrating that it reduces cloud usage while maintaining latency deadline satisfaction compared to baseline strategies.

The remainder of this paper is organized as follows. Section 2 reviews related work in learning-based multi-view 3D reconstruction. Section 3 presents the considered multi-stage reconstruction pipeline and discusses its computation and communication characteristics. Section 4 introduces the system model and formulates the cost-aware scheduling problem under latency constraints, while Section 5 presents the proposed online scheduling algorithm. Section 6 evaluates the proposed approach using both real-system measurements and simulation experiments. Finally, Section 7 concludes the paper and presents future work.

## 2. Related Work

Multi-view 3D reconstruction has been extensively studied across the computer vision and systems communities, with research spanning geometric pipelines, deep learning methods, and system-level optimizations for real-world deployment.

### 2.1. Traditional and Learning-Based Multi-View Reconstruction

Traditional multi-view reconstruction approaches rely on geometric pipelines such as Structure-from-Motion (SfM) and multi-view stereo (MVS), which reconstruct a 3D structure by establishing correspondences across multiple images. While effective, these methods are computationally intensive and require careful calibration, limiting their applicability in latency-sensitive environments [4].

With the emergence of deep learning, learning-based methods have improved the robustness and efficiency of multi-view reconstruction. These methods typically follow a modular design comprising feature extraction, view fusion, and 3D decoding, but they often treat these components independently, thereby limiting their ability to capture complex relationships across views [5].

To address these limitations, transformer-based architectures have recently been introduced. Wang et al. [5] propose a unified framework (VolT) that reformulates multi-view 3D reconstruction as a sequence-to-sequence prediction problem. By leveraging self-attention, the model jointly processes multiple views and captures their relationships more effectively. Several practical implementations of this idea have been proposed, including MVSFormer [9], which applies transformer-based multi-view stereo, and MapAnything [10], a transformer-based model for metric 3D reconstruction from arbitrary input configurations, including calibrated and uncalibrated image collections as well as inputs augmented with camera poses or depth information.

### 2.2. Latency-Aware and Edge-Based Reconstruction

As multi-view reconstruction is increasingly deployed in real-world applications, latency becomes a critical concern. Zhang et al. [4] show that traditional SfM+MVS pipelines are too slow for real-time operation in edge environments, particularly in disaster response scenarios. To address this, they propose a collaborative mobile edge computing (MEC) framework that introduces both data-level and task-level parallelism. By decomposing reconstruction tasks and distributing them across edge nodes, their approach achieves significant latency reductions while preserving the reconstruction quality.

Other relevant solutions have focused explicitly on balancing latency and reconstruction quality in traditional multi-view reconstruction pipelines. In particular, ref. [7] formulates the problem of edge-native multi-view reconstruction as a trade-off between accuracy and responsiveness. Their approach dynamically adjusts parameters such as the input resolution, view selection, and computational allocation to satisfy latency constraints while maintaining acceptable reconstruction quality. Similarly, edge-assisted systems such as Edge-SLAM [11] offload parts of the reconstruction pipeline to edge resources to reduce processing delays while maintaining reconstruction quality.

Recent works explore adaptive edge resource management techniques. Reinforcement learning has been proposed as a mechanism for dynamically scheduling reconstruction tasks under varying system conditions. For example, ref. [8] introduces a reinforcement learning-based framework for edge resource management in multi-view 3D reconstruction. The approach learns scheduling policies that adapt to changing workloads, network conditions, and device heterogeneity, enabling improved reliability and performance. While such RL-based approaches provide flexibility and adaptability, they often require training and may introduce additional system complexity, making them less predictable in latency-critical applications.

More generally, workflow scheduling in the edge–cloud continuum has been studied from a multi-objective optimization perspective. Zanussi et al. [12] formulate workflow placement as a trade-off between execution costs and latency and propose a simulation-driven optimization framework for identifying efficient deployment configurations. While such approaches effectively explore the cost–latency trade-off, they do not explicitly enforce latency SLOs and are primarily designed for offline optimization. More recently, Cao et al. proposed LASSY, a latency-aware SLO-sufficing scheduler for cloud–edge environments that combines queuing-theoretic latency modeling with cost-aware resource allocation [13]. LASSY predicts tail latency and determines service placements that satisfy latency SLOs while minimizing operational costs. However, the approach targets monolithic latency-sensitive services and optimizes service placement at the application level rather than scheduling individual stages of data-intensive processing pipelines. In contrast, our work focuses on learning-based multi-view 3D reconstruction pipelines, where placement decisions must additionally account for stage-level computation costs, large intermediate data transfers, and communication-aware pipeline partitioning.

### 2.3. Summary and Research Gap

In summary, prior work has focused on improving reconstruction quality through traditional and learning-based methods, reducing latency through edge-assisted execution, and optimizing service placement through cost-aware or SLO-aware scheduling approaches. However, these works either target traditional reconstruction pipelines, optimize monolithic services, or consider workflow scheduling independently of the characteristics of modern learning-based reconstruction pipelines. Despite these advances, there is a gap in cloud cost-aware scheduling under latency constraints for learning-based multi-view 3D reconstruction pipelines deployed across the edge–cloud continuum. This work integrates a learning-based reconstruction pipeline with an online scheduling framework that explicitly considers the computational cost, communication overhead, and resource contention to reduce cloud resource usage while satisfying latency constraints.

## 3. Learning-Based Multi-View 3D Reconstruction Pipeline

**Figure 1:**
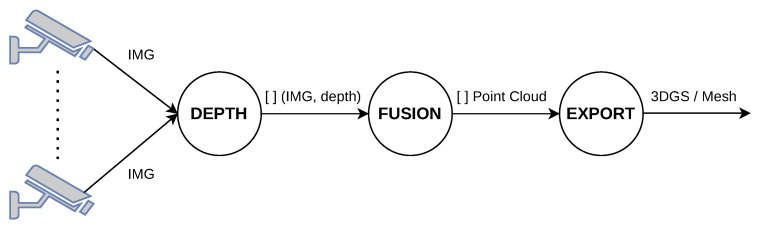
Pipeline for learning-based multi-view 3D reconstruction.

Modern multi-view 3D reconstruction has increasingly shifted from traditional geometry-based approaches to learning-based pipelines that leverage deep neural networks to infer scene structures from images [5,14]. These pipelines combine learned models with geometric reasoning, enabling more robust reconstruction in challenging conditions and reducing the reliance on precise calibration. In this work, we consider a modular pipeline composed of three stages: depth estimation, transformer-based multi-view fusion, and point cloud generation with rendering preparation. An overview of the pipeline is shown in Figure 1. The system takes as input a set of images captured from different viewpoints and progressively transforms them into a unified 3D representation.

### 3.1. Pipeline Stages

The first stage (*DEPTH*) performs per-view depth estimation. Given a batch of RGB images, the model processes each image independently and predicts dense depth maps, optionally including geometric information such as ray directions or camera intrinsics. The output is a set of per-pixel depth representations, which form the first geometry-aware representation of the scene. Due to their high resolution and per-view nature, these representations are relatively large. Depending on the deployment strategy, this stage can be executed either as a single task processing the entire image batch or as multiple parallel tasks distributed across different nodes to improve system performance.

The second stage (*FUSION*) performs multi-view fusion using a transformer-based model. It takes as input the images together with their corresponding depth maps and geometric information, and it jointly processes all views to infer a coherent 3D structure of the scene. Unlike the depth stage, this stage jointly processes multiple views to aggregate complementary information and enforce geometric consistency. The output consists of 3D point predictions with associated confidence values, representing a unified multi-view reconstruction. Depending on the model architecture and representation format, the resulting representation can be smaller than the dense depth outputs due to multi-view aggregation and intermediate downsampling.

The final stage (*EXPORT*) converts the multi-view predictions into a unified 3D representation and prepares it for efficient rendering. It aggregates and filters the predicted 3D points and exports them into a rendering-friendly format, such as 3D Gaussian splats [15] or meshes [16], to enable efficient visualization and downstream processing. The size and structure of the final representation depend on the selected rendering format. Representations based on 3D Gaussian splats can provide relatively compact scene encoding through compressed point-based primitives, whereas mesh-based representations may become substantially larger due to additional connectivity and triangulation information between vertices. Consequently, the final rendering-oriented representation is often the largest data representation in the pipeline. This stage produces the final structured representation of the reconstructed scene, completing the transformation from raw images to a 3D output.

### 3.2. Stage Placement Considerations

The pipeline structure shown in Figure 1 directly affects stage placement decisions across the edge–cloud continuum. In particular, the size and structure of intermediate representations can vary significantly across stages. The depth stage typically produces large outputs due to the dense per-pixel representations generated for each input image. In contrast, the fusion stage may produce smaller intermediate representations due to multi-view aggregation, filtering, or downsampling, whereas the final rendering-oriented representation can again increase in size depending on the selected format.

These differences directly affect the communication cost when stages are executed on different nodes. Consequently, the communication overhead depends strongly on where the pipeline is partitioned and which intermediate representation is transferred between stages. From the computational perspective, the model and export stages are the most computationally intensive, while the depth stage has a relatively low processing cost. Therefore, the placement of pipeline stages has a critical impact on end-to-end latency, as it requires balancing computation and communication. This trade-off is a well-known challenge in edge–cloud systems [17,18]. This motivates the need for scheduling strategies that jointly consider the processing time, data transfer cost, and system dynamics when distributing the pipeline across edge and cloud resources.

## 4. System Model and Problem Formulation

We consider a multi-view 3D reconstruction system deployed across an edge–cloud continuum. A set of geographically distributed sources (e.g., drones) continuously capture images and generate 3D reconstruction requests. Each request consists of a batch of synchronized images that are processed through a multi-stage pipeline.

The system comprises

- A set of source nodes S generating requests;
- A set of edge nodes E, each characterized by limited computational capacity;
- A cloud node *c* with virtually unlimited computational resources.

The nodes are interconnected by links with finite capacity. The available bandwidth between nodes *i* and *j* is denoted as $B_{ij}$.

**Figure 2:**
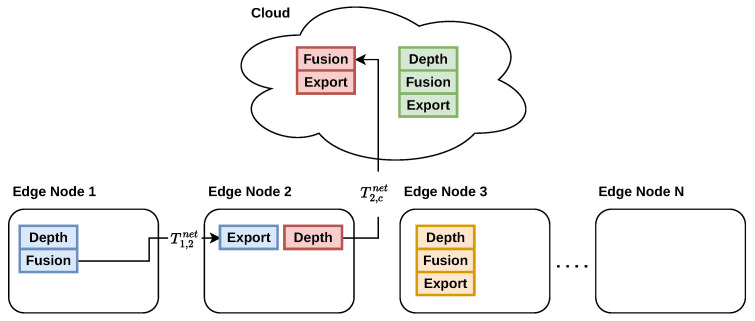
Edge–cloud system model for multi-view 3D reconstruction.

Figure 2 illustrates the considered edge–cloud system, which consists of a set of edge nodes and a cloud node. Each node is capable of executing any stage of the reconstruction pipeline, including multiple stages simultaneously. When consecutive pipeline stages are assigned to different nodes, the resulting intermediate data must be transmitted over the network; this incurs communication latency, determined by the data volume and the available bandwidth between the nodes.

### 4.1. Pipeline Model

Each reconstruction request is processed through a fixed sequence of three stages:

depth→fusion→export.

For a request *r*, each stage must be assigned to a compute node. A placement is defined as

(1) $$p=(n_{d},n_{f},n_{e}),$$

where $n_{d},n_{f},n_{e}\in E\cup \left\{c\right\}$ denote the nodes executing the depth, fusion, and export stages, respectively.

Each stage has a processing time that depends on the selected node:

(2) $$T_{k}\left(n\right),\;k\in \left\{d,f,e\right\}.$$

### 4.2. Data Transfer Model

Data are transferred between pipeline stages if they are executed on different nodes. The transfer time between nodes *i* and *j* is

(3) $$T_{ij}^{\mathrm{net}}\left(M\right)=\frac{M}{B_{ij}},$$

where *M* is the size of the transmitted data.

The pipeline involves three data transfers:

- Raw input images from source to depth node;
- Depth outputs from depth node to fusion node;
- Point cloud outputs from fusion node to export node.

### 4.3. Streaming Workload Model

Requests are generated periodically by each source. For each pipeline stream *i*, requests arrive at times

(4) $$t_{i}^{k}=\phi_{i}+k\cdot P_{i},$$

where $P_{i}$ is the period and $\phi_{i}$ is the phase offset. Multiple streams may execute concurrently, sharing network and compute resources.

### 4.4. Resource Model

Edge nodes are modeled as single-server systems, where tasks are processed sequentially. Each edge node *e* maintains a readiness time $R_{e}$, representing when it becomes available. Similarly, each directed network link (i,j) maintains a readiness time $R_{ij}$ to model link contention. The cloud is modeled as an elastic resource with effectively unlimited processing capacity and no queuing delay. This assumption is commonly used in edge–cloud systems, where cloud providers can provision computational resources on demand, while resource contention is primarily expected at capacity-constrained edge nodes [19].

### 4.5. Latency Model

Given a request *r* arriving at time $t_{r}$ and placement $p=(n_{d},n_{f},n_{e})$, the completion time is determined by sequentially accounting for (i) communication delays, (ii) queuing delays due to resource contention, and (iii) processing times.

The end-to-end latency is

(5) $$L\left(r,p\right)=t_{\mathrm{finish}}\left(r,p\right)-t_{r}.$$

### 4.6. Service Level Objective (SLO)

Each request must satisfy a latency deadline,

(6) L(r,p)≤D,

where *D* is a predefined SLO.

### 4.7. Cost Model

Cloud execution incurs a monetary cost proportional to the usage time. Since cloud pricing is linear with the execution time in our model, the cost is represented using equivalent cloud execution time units. The cost of executing request *r* under placement *p* is

(7) $$C\left(r,p\right)=\sum_{k\in K}I\left[n_{k}=c\right]\cdot T_{k}\left(c\right),$$

where I[·] is the indicator function.

### 4.8. Problem Formulation

At each request arrival, the scheduler must select a placement that minimizes the cloud cost while satisfying the latency constraint:

(8) $$\begin{array}{cc} \min_{p}\; & C(r,p) \end{array}$$

(9) $$\begin{array}{cc} s.t.\; & L(r,p)\leq D. \end{array}$$

This defines an online decision problem where scheduling decisions are made sequentially based on the current system state.

## 5. Online SLO-Aware Scheduling Algorithm

We propose an online scheduling algorithm for multi-view 3D reconstruction pipelines that assigns pipeline stages to compute nodes upon each request arrival. The scheduler is state-aware and considers both current node occupancy and the network conditions. The algorithm outline is shown in Algorithm 1.

To improve its scalability, the algorithm reduces the placement search space by selecting only the most promising edge nodes for each request. This is achieved through a top-*K* edge selection step, which ranks edge nodes using a score that combines the transfer delay and queuing delay. Candidate placements are then generated only from the selected top-*K* edge nodes and the cloud node. The algorithm follows a greedy strategy: it selects the lowest-cost placement that satisfies the latency constraint and falls back to the minimum-latency placement in the cloud if no feasible candidate exists.

### 5.1. Top-K Edge Selection

Let *r* denote a request arriving at time $t_{r}$ from source node *s*. For each edge node e∈E, the scheduler computes the score

(10) $$\mathrm{score}\left(e\right)=T_{s,e}^{\mathrm{net}}\left(M_{\mathrm{img}}\right)+\max\left(0,R_{e}-t_{r}\right),$$

where

- $T_{s,e}^{\mathrm{net}}\left(M_{\mathrm{img}}\right)$ is the time required to upload the raw input images from source *s* to edge node *e*;
- $R_{e}$ is the current readiness time of edge node *e*;
- $t_{r}$ is the release time of request *r*.

**Algorithm 1** Online Cheapest-Feasible Scheduling with Top-*K* Edge Pruning
**Require:** 
Request *r* with source *s* and release time $t_{r}$**Require:** 
Edge set E, cloud node *c*, deadline *D***Require:** 
Current node readiness times and link readiness times1:Compute score(e) for each e∈E using Equation (10)2:Select the top-*K* edge nodes with the smallest scores3:Construct candidate set $P_{K}$ from the selected edge nodes and cloud node4:Sort candidates in $P_{K}$ by increasing cloud cost5:

$p_{\mathrm{best}}\leftarrow ⌀$

6:

$p_{\mathrm{fallback}}\leftarrow ⌀$

7:**for** each placement $p\in P_{K}$ **do**8:    Compute nominal latency $L_{\mathrm{nominal}}\left(p\right)$9:    **if** $L_{\mathrm{nominal}}\left(p\right)>D$ **then**10:        Predict latency L(p) using current resource state11:        **if** $p_{\mathrm{fallback}}=⌀$ or $L\left(p\right)<L\left(p_{\mathrm{fallback}}\right)$ **then**12:           $p_{\mathrm{fallback}}\leftarrow p$13:        **end if**14:        **continue**15:    **end if**16:    Predict latency L(p) using state-aware simulation17:    **if** L(p)≤D **then**18:        $p_{\mathrm{best}}\leftarrow p$19:        **break**20:    **else**21:        **if** $p_{\mathrm{fallback}}=⌀$ or $L\left(p\right)<L\left(p_{\mathrm{fallback}}\right)$ **then**22:              $p_{\mathrm{fallback}}\leftarrow p$23:        **end if**24:    **end if**25:
**end for**
26:**if** $p_{\mathrm{best}}\neq ⌀$ **then**27:    Commit $p_{\mathrm{best}}$ by updating node and link readiness times28:    **return** $p_{\mathrm{best}}$29:
**else**
30:    Commit $p_{\mathrm{fallback}}$31:    **return** $p_{\mathrm{fallback}}$32:
**end if**



The first term captures network proximity, while the second term captures the waiting time before depth execution can start on the edge node. Thus, the score estimates the earliest time at which the request could begin processing on edge node *e*.

The scheduler sorts all edge nodes by this score and selects the top-*K* with the lowest values (lines 1 and 2). The cloud node is then added unconditionally, ensuring that a feasible fallback is always available. The choice of *K* depends on the characteristics of the edge–cloud topology, particularly the density and distribution of edge nodes. In practice, sources are typically connected to only a small subset of nearby edge nodes with favorable network conditions, making small values of *K* sufficient to capture the most relevant placement candidates while significantly reducing the search space.

### 5.2. Candidate Placement Construction

After selecting the top-*K* edge nodes, the scheduler constructs the candidate set of placements as

(11) $$P_{K}=\left\{\left(n_{d},n_{f},n_{e}\right)|n_{d},n_{f},n_{e}\in E_{K}\cup \left\{c\right\}\right\},$$

where $E_{K}\subseteq E$ is the set of the top-*K*-ranked edge nodes. The resulting candidate space contains at most ${\left(K+1\right)}^{3}$ placements, which is substantially smaller than the full search space of $N^{3}$, where N=|E|+1 is the total number of compute nodes including the cloud. Candidates are then sorted in ascending order regarding the cloud execution cost.

### 5.3. State-Aware Evaluation

For each candidate placement $p=(n_{d},n_{f},n_{e})$, the scheduler performs two levels of evaluation (lines 7–25). First, it computes a nominal latency, which ignores queuing effects:

(12) $$L_{\mathrm{nominal}}\left(p\right).$$

If this nominal latency already exceeds the deadline *D*, the placement is discarded from feasibility consideration, since contention can only increase the end-to-end delay.

Second, for the remaining candidates, the scheduler predicts the actual completion time by simulating execution under the current resource state. This prediction considers

- the current readiness times of edge nodes;
- the current readiness times of network links;
- the stage execution times;
- the data transfer times between stages.

### 5.4. Placement Selection

The scheduler evaluates placement candidates in increasing order of cloud cost and selects the cheapest candidate whose predicted latency satisfies the deadline; if no such candidate exists, it selects the one with the minimum predicted latency. Once a placement is selected, the scheduler immediately commits it by updating the readiness times of the involved nodes and links.

### 5.5. Complexity Analysis

Let E=|E| denote the number of edge nodes and K≤E be the number of selected edge nodes per decision. The scheduler performs two main steps.

- Edge ranking: All edge nodes are scored and sorted, with complexity
  (13) O(ElogE).
- Candidate evaluation: Placements are generated from the selected top-*K* edge nodes and the cloud, resulting in ${\left(K+1\right)}^{3}$ candidates. Since each candidate evaluation touches only a constant number of resources, this contributes
  (14) $$O({\left(K+1\right)}^{3}).$$

Therefore, the per-request complexity of the proposed scheduler is

(15) $$O(E\log E+{\left(K+1\right)}^{3}).$$

This improves over the full-search baseline complexity of $O(E^{3})$ while retaining state-aware decision-making. In practice, *K* is typically much smaller than *E*, since only a limited subset of edge nodes is usually close to a given source in terms of network conditions. As a result, the scheduler can significantly reduce the search space while remaining suitable for real-world deployment.

## 6. Experimental Evaluation

We evaluate the proposed scheduling approach in the context of a disaster response use case, where multiple drones capture images of the same scene from different viewpoints and a multi-view 3D reconstruction pipeline is executed in near-real time to provide situational awareness for emergency operators [4].

The evaluation consists of three parts. First, we implement a full learning-based 3D reconstruction pipeline composed of depth estimation, multi-view fusion, and point cloud merging with 3D Gaussian Splatting export. Second, we experimentally characterize the performance of the pipeline on real hardware under different edge–cloud deployment configurations. Third, we use these measurements to parameterize a simulator and evaluate the proposed scheduler under larger-scale workloads.

### 6.1. Prototype Pipeline Implementation

We implemented a modular three-stage pipeline (source code: https://github.com/cilicivan/multiview-3d-rec, accessed on 6 July 2026) consisting of the following:

- Depth estimation: per-view depth estimation using UniK3D [20];
- Multi-view fusion: transformer-based reconstruction using MapAnything [10]; and
- Merging and splatting: point cloud merging and 3D Gaussian splatting export.

The prototype is implemented as a unified service exposing three HTTP endpoints: /depth, /model, and /splat. Each stage can either execute locally on the current host or forward its output to another host for the execution of the subsequent stage. This behavior is controlled through query parameters, allowing flexible execution chains without modifying the pipeline logic itself.

The client submits a batch of input images to the /depth endpoint as a multipart HTTP request. The depth service reads and resizes the input images, performs batched inference with the UniK3D monocular depth estimator, and produces per-view depth predictions together with geometric information required for the next stage. Depending on the configuration, the output is either passed directly in-process to the fusion stage or serialized into an NPZ payload and sent to a remote /model endpoint for multi-view fusion.

The multi-view fusion stage consumes the images together with the depth outputs and runs MapAnything [10], a transformer model for feed-forward metric 3D reconstruction from calibrated or uncalibrated multi-view observations, to infer the aligned 3D scene structure. Its outputs include per-view 3D points, confidence values, and auxiliary camera-related information. As with the depth stage, the results can either be used locally by the splatting stage or serialized and forwarded over HTTP to another host.

The final stage receives the 3D outputs of the fusion stage, merges them into a unified point cloud, applies filtering and downsampling, and exports the result in a 3D Gaussian splatting-compatible PLY format. Internally, this stage is implemented using Open3D [21] and a conversion procedure that prepares point attributes such as scale, opacity, and spherical harmonics coefficients required for Gaussian splatting.

The implementation also distinguishes between GPU-bound and CPU-bound parts of the pipeline. The depth and fusion stages are executed on the GPU, while the final merging and export stage is primarily CPU- and memory-bound. To avoid uncontrolled contention on the GPU, inference requests are serialized using a single-worker execution pool on each host. In contrast, CPU-heavy operations such as NPZ serialization and point cloud export are handled separately. This design is important for the evaluation, as it ensures that the measured latencies reflect realistic per-stage execution behavior under service-based deployment.

Finally, the service records detailed timing information for each stage and inter-stage transmission. For each request, the measured values include the depth inference time, model inference time, splatting time, upload time between depth and model when the stages are placed on different hosts, and upload time between model and splat when applicable. These measurements are later used to derive the simulator parameters and to analyze the computation–communication trade-offs of different pipeline placements.

### 6.2. Real-System Performance Evaluation

To characterize the performance of the proposed reconstruction pipeline in an edge–cloud environment, we deploy the pipeline on representative edge and cloud hardware platforms. The edge platform is represented by an NVIDIA AGX Orin 64 GB device equipped with an onboard GPU (Santa Clara, CA, USA), while the cloud platform consists of a virtual machine with 16 CPU cores, 120 GB RAM, and an NVIDIA A100 40 GB GPU. The goal of this evaluation is to measure the execution and communication characteristics of individual pipeline stages under realistic deployment conditions and to use these measurements to parameterize the simulator used in the later large-scale evaluation.

The experiments are performed using a single pipeline instance without competing workloads or resource sharing between requests. This allows us to isolate the execution characteristics of each stage and measure the baseline processing and transfer costs of the pipeline. Input images are full HD JPEGs, where a batch of four images has a total size of 2.6 MB. The corresponding intermediate representations vary significantly across stages: the depth stage produces approximately 55.4 MB of output data and the transformer-based fusion stage produces 16.3 MB (due to downsampling), while the final export stage produces a Gaussian splat representation of approximately 120 MB.

To evaluate the impact of stage placement, we consider four scheduling configurations:

- E–E–E, where all pipeline stages are executed on the edge node;
- E–E–C, where the depth and fusion stages are executed on the edge, while the export stage is offloaded to the cloud;
- E–C–C, where only the depth stage is executed on the edge, while the fusion and export stages are executed in the cloud;
- C–C–C, where all pipeline stages are executed in the cloud.

#### 6.2.1. Overall Performance Across Configurations

**Figure 3:**
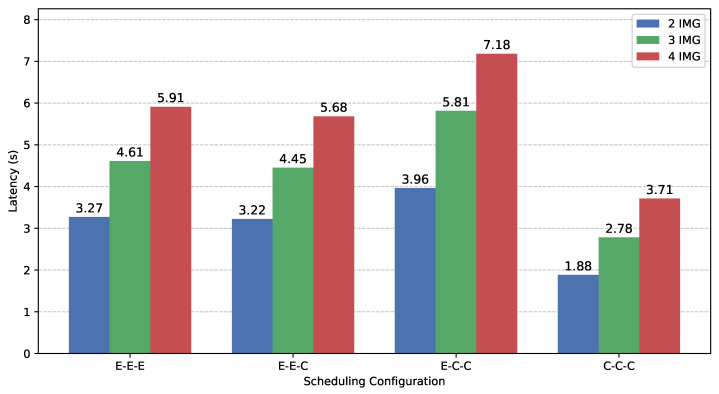
Overall pipeline latency for different deployment configurations and numbers of input images.

Figure 3 shows the overall latency across all deployment configurations and input sizes (two, three, and four images).

Several important trends can be observed. First, the fully cloud-based deployment (C–C–C) consistently achieves the lowest latency for all input sizes, with measured end-to-end delays of 1.88 s, 2.78 s, and 3.71 s for two, three, and four images, respectively. This confirms that the cloud platform provides significantly higher processing throughput for all pipeline stages.

Second, the fully edge-based deployment (E–E–E) exhibits the highest computation time among configurations without offloading, reaching 5.91 s for four images. This reflects the substantially lower processing capabilities of the edge device, especially for GPU-intensive stages.

Third, the two hybrid deployments behave differently. The E–E–C configuration remains close to the all-edge case for smaller image batches, but it becomes slightly faster than all-edge deployment for the four-image case. In contrast, the E–C–C configuration becomes the slowest configuration at larger batch sizes, reaching 7.18 s for four images. This already suggests that the placement boundary between depth and model is particularly expensive and that the communication cost can outweigh the computational gains of moving later stages to the cloud.

#### 6.2.2. Representative Case: Four-Image Pipeline

**Figure 4:**
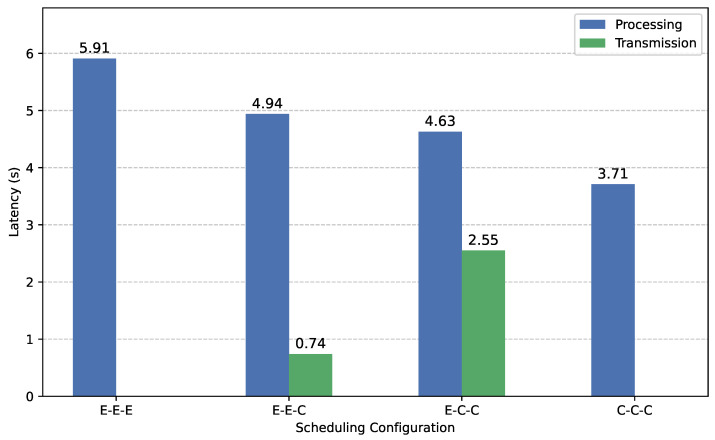
Processing and transmission latency for the four-image pipeline.

We focus on the four-image case as it represents the most computationally demanding scenario supported on the edge platform. Figure 4 shows the processing and transmission latency, and Table 1 presents a detailed breakdown for each pipeline stage.

The results provide several insights relevant to the scheduling problem. From a computation perspective, cloud execution significantly accelerates the depth and fusion stages, confirming the advantage of offloading computationally intensive processing to more powerful cloud resources. However, the communication overhead between stages is highly asymmetric due to the varying sizes of intermediate representations. In particular, transferring the large depth-stage output incurs substantially higher latency than transferring the smaller fusion-stage representation, making early-stage offloading considerably more expensive. Consequently, the E–E–C configuration emerges as the most effective hybrid deployment among those evaluated, as it avoids the costly depth-to-model transfer while still offloading the computationally intensive export stage to the cloud. These results highlight the importance of jointly considering both computation and communication when determining stage placement across the edge–cloud continuum.

### 6.3. Simulation-Based Scheduler Evaluation

To evaluate the proposed scheduling approach under larger-scale workloads, we implemented a discrete event simulator (source code and experimental results: https://github.com/cilicivan/3d-rec-scheduler, accessed on 6 July 2026) parameterized using the measured performance results. The simulator models

- periodic pipeline requests;
- edge nodes with queuing;
- cloud nodes with unlimited capacity;
- network links with finite bandwidth.

The simulator uses the measured four-image execution times and intermediate data sizes, since this case is the most representative and the most demanding one supported by the edge platform. Each request is generated by a source node and processed through the same three-stage pipeline as in the real implementation.

#### 6.3.1. Simulation Setup

We consider three workload scenarios with 10, 15, and 20 pipeline streams, while keeping the infrastructure fixed at five edge nodes and one cloud node. These scenarios are designed to evaluate the impact of the pipeline-to-edge-node ratio on the scheduling behavior and resource utilization. As the number of concurrent pipelines increases relative to the available edge resources, edge-node contention and queuing delays become more pronounced, reducing the scheduler’s ability to keep computation at the edge, which in turn increases the reliance on cloud execution.

The latency deadline is set to 6 s, which represents a realistic near-real-time target for multi-view reconstruction applications [4] and is also consistent with the stage latencies observed in the real-system experiments.

**Figure 5:**
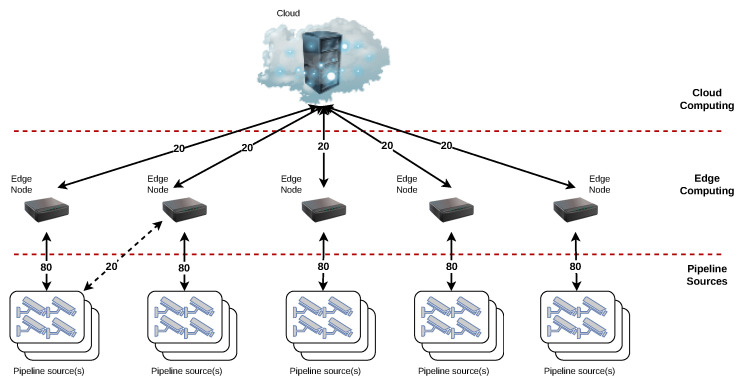
Simulated topology used in the evaluation. Arrows represent communication links, with labels indicating the available bandwidth between connected nodes.

The scheduler is evaluated across three workload scenarios with different network topologies. An example topology is shown in Figure 5, illustrating the pipeline sources represented by four cameras, five edge nodes, the cloud node, and the bandwidth links between them. (The bandwidth values used in the simulations (80 MB/s and 20 MB/s) are roughly based on transfer rates measured in our local network and between the local network and the cloud node used in the real-system experiments.)

#### 6.3.2. Comparison with Baselines

We compare the proposed scheduler against two baseline strategies that represent the extreme points of the edge–cloud design space.

- Online Scheduler (proposed): An online scheduler (Algorithm 1) that dynamically selects the execution location of each pipeline stage (depth, fusion, export) for every incoming request. At runtime, it evaluates candidate placements across edge and cloud nodes using the proposed top-*K* edge selection strategy (K=2 in this evaluation) and chooses the lowest-cost configuration that satisfies the latency deadline.
- Pareto-SLO: A baseline inspired by the workflow scheduling framework of Zanussi et al. [12]. For each request, the scheduler evaluates all possible stage placements and identifies the Pareto front with respect to the cloud cost and latency. Among the Pareto-optimal placements, it selects the lowest-cost configuration that satisfies the latency deadline. Unlike the proposed scheduler, Pareto-SLO performs an exhaustive search over the placement space and therefore represents a higher-complexity scheduling approach.
- Edge-Greedy: A baseline that attempts to execute the entire pipeline on a single edge node, without splitting stages across multiple nodes. For each request, the scheduler evaluates all edge nodes by assigning the depth, fusion, and export stages to the same node. If at least one edge node can satisfy the latency deadline, the request is scheduled on the edge node with the lowest predicted latency. Otherwise, the entire pipeline is offloaded to the cloud. This strategy prioritizes edge execution and avoids intermediate transfers between different compute nodes, but it may still rely on the cloud when edge execution cannot satisfy the latency constraint.
- Cloud-Only: A baseline that executes all pipeline stages in the cloud. This eliminates edge resource constraints and avoids inter-stage communication costs, resulting in the lowest latency. However, it incurs the highest cloud resource usage and therefore represents the upper bound on the operational cost.

**Figure 6:**
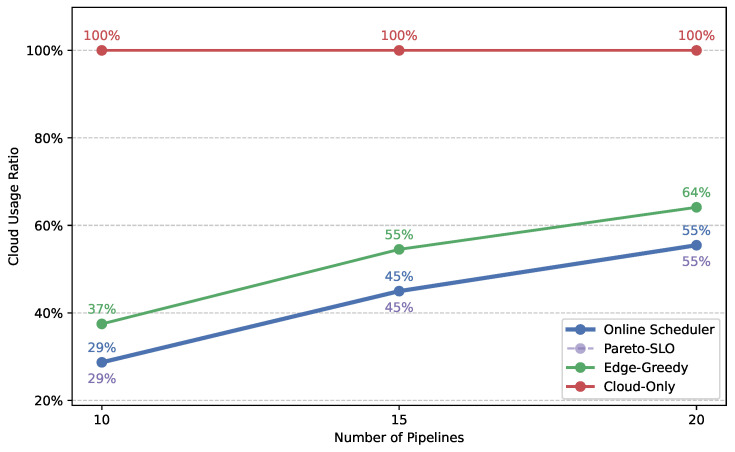
Cloud usage ratio for different scheduling strategies.

The first evaluation metric is the cloud cost, shown in Figure 6, as the main objective of the proposed scheduler is to reduce cloud resource utilization while satisfying the latency constraint. The proposed scheduler and Pareto-SLO achieve identical cloud costs across all scenarios, indicating that the proposed scheduler is able to identify placements comparable to those obtained through exhaustive Pareto-front exploration. Both approaches substantially reduce the cloud cost compared to the Cloud-Only baseline, achieving reductions of approximately 50–70% depending on the scenario. As expected, the largest improvement is observed in the 10-pipeline scenario, where the ratio of edge resources to pipelines is the most favorable (two pipelines per edge node), allowing a larger fraction of requests to remain fully or partially at the edge. As the number of pipelines increases to 15 and 20, edge-node contention becomes more pronounced, increasing the need for cloud offloading. Compared to the Edge-Greedy baseline, both approaches further reduce the cloud cost by exploiting mixed edge–cloud placements and distributing pipeline stages across multiple resources instead of forcing the entire pipeline onto a single node.

**Figure 7:**
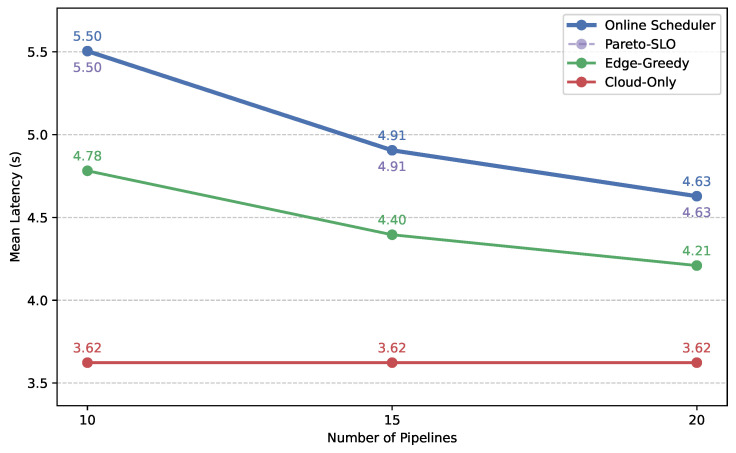
Average latency comparison across scheduling strategies.

Figure 7 shows the corresponding average end-to-end latency. The proposed scheduler and Pareto-SLO achieve identical latency, further confirming that the proposed top-*K* search identifies placements that are close to those obtained through exhaustive Pareto-front exploration. However, both approaches exhibit higher latency than Cloud-Only and Edge-Greedy. This is expected, as the scheduler maximizes the use of edge resources and may introduce additional transmission overhead when stages are distributed across nodes, as discussed in Section 4. Nevertheless, the primary objective is not to minimize the latency but rather to satisfy the latency deadline while reducing the cloud cost. Importantly, all evaluated strategies achieved full deadline satisfaction across all scenarios, meaning that all requests satisfied the 6 s latency constraint.

**Figure 8:**
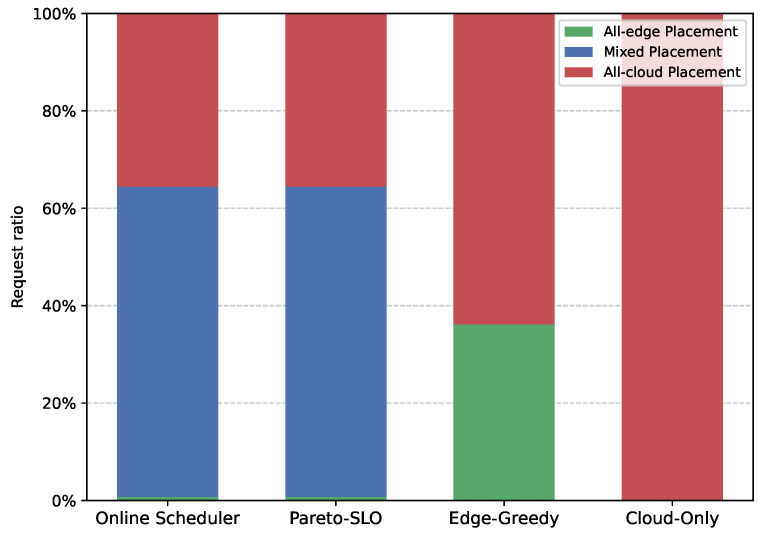
Placement approach ratio across scheduling strategies.

Figure 8 shows the ratio of placement approaches used by each scheduling strategy, distinguishing between fully edge-based, fully cloud-based, and mixed edge–cloud execution. The figure highlights the fundamental difference between the proposed scheduler and the Edge-Greedy baseline. The proposed scheduler primarily favors mixed placements, where different pipeline stages are distributed across edge and cloud resources. This enables the more efficient utilization of edge capacity by offloading only the most computationally intensive stages when necessary. In contrast, the Edge-Greedy strategy cannot split the pipeline and therefore attempts to execute the entire pipeline on a single edge node whenever possible. As a result, edge resources become occupied for longer periods, increasing queue buildup and forcing more requests to be fully offloaded to the cloud.

##### Comparison with Pareto-SLO Scheduling

While the previous results show that the proposed scheduler achieves a cloud cost and latency comparable to those of Pareto-SLO, the two approaches differ significantly in scheduling complexity. Pareto-SLO evaluates the complete placement space for each incoming request in order to identify Pareto-optimal solutions, resulting in up to ${\left(N+1\right)}^{3}$ candidate placements. In contrast, the proposed scheduler first restricts the search space using the top-*K* edge selection mechanism and therefore evaluates at most ${\left(K+1\right)}^{3}$ candidates. Although both schedulers produce nearly identical cloud costs, latencies, and placement decisions, the average scheduling latency in the evaluated scenario was 2.86 ms for Pareto-SLO, compared to only 0.11 ms for the proposed scheduler. These results indicate that the proposed scheduler achieves placement quality comparable to that of exhaustive Pareto-front exploration while maintaining substantially lower computational overhead, making it more suitable for online scheduling in dynamic edge–cloud environments.

#### 6.3.3. Impact of the Top-K Parameter

The proposed scheduler reduces the search space by considering only the top-*K* edge nodes when generating candidate stage placements. The rationale behind this design is to balance scheduling quality against the decision-making overhead. A larger value of *K* increases the number of candidate placements evaluated by the scheduler, potentially improving placement quality but also increasing the scheduling complexity.

In the evaluation scenarios presented so far, all edge nodes were assumed to have identical processing capabilities. Under such conditions, the top-*K* parameter has little impact on the scheduling quality. Since all edge nodes exhibit the same execution latency, the node-ranking procedure effectively orders nodes according to their predicted availability and communication latency. Consequently, the highest-ranked node is typically also the best execution choice, and considering additional edge nodes provides little additional benefit.

To better understand the effect of *K*, we constructed an additional heterogeneous edge scenario. The topology and workload configuration remained unchanged, consisting of five edge nodes, one cloud node, and twenty pipeline streams. However, two of the five edge nodes were assumed to provide approximately 30% lower execution latency for all pipeline stages, representing a more powerful edge-AI platform, such as the NVIDIA Jetson AGX Thor. This creates a non-uniform edge environment in which the node-ranking decision becomes more important.

**Figure 9:**
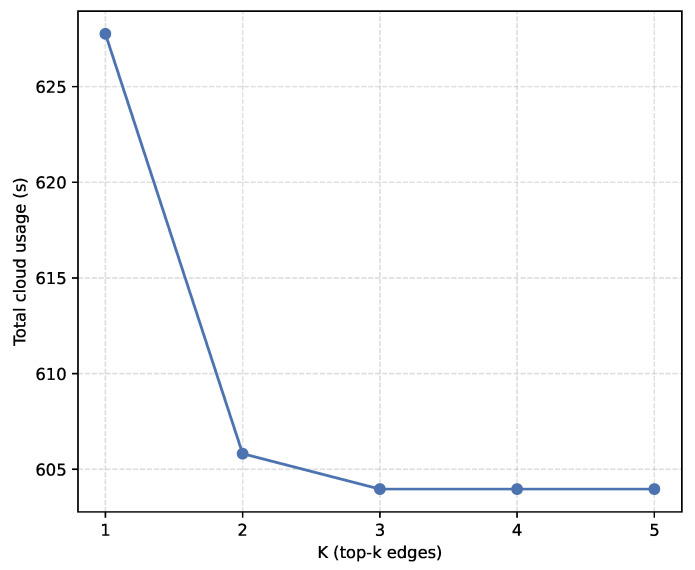
Cloud cost as a function of the top-*K* parameter.

Figure 9 shows the resulting cloud cost for different values of *K*. We observe that increasing *K* from one to two noticeably improves the scheduling quality, as the scheduler gains access to a larger set of candidate placements and is more likely to exploit the faster edge nodes. However, further increases in *K* provide only marginal improvements. In particular, the results indicate that K=2 already achieves a solution that is very close to the best placement quality obtained with larger values of *K*, suggesting that only a small number of candidate edge nodes are required to capture most of the potential benefit.

**Figure 10:**
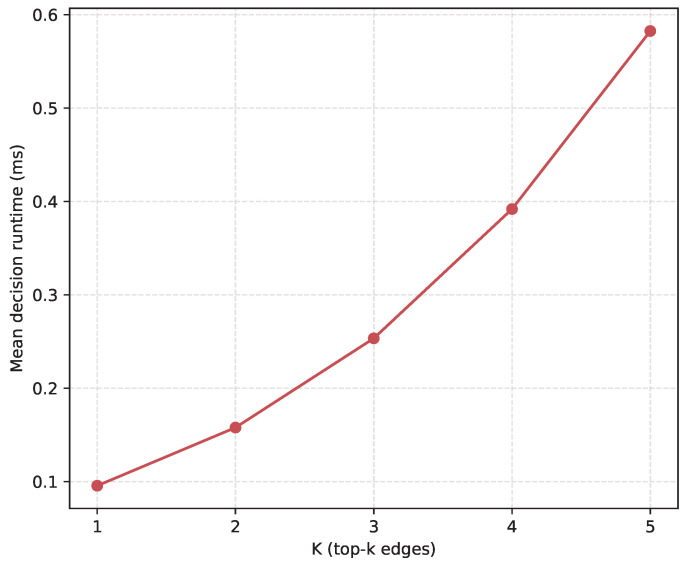
Average scheduler decision latency as a function of the top-*K* parameter.

Figure 10 shows the corresponding scheduler runtime. As expected, the decision latency increases rapidly with larger values of *K*. This behavior follows directly from the candidate generation procedure, which evaluates up to ${\left(K+1\right)}^{3}$ stage placement combinations. Consequently, each increment in *K* expands the search space significantly, resulting in an exponential increase in scheduling overhead.

Overall, the results show that a small value of *K* provides an effective trade-off between scheduling quality and decision overhead. In the evaluated scenarios, K=2 captures nearly all achievable cloud cost savings while maintaining very low scheduling latency, which justifies its use throughout the remainder of the evaluation.

## 7. Conclusions and Future Work

In this paper, we address the problem of cost-aware scheduling under latency constraints for learning-based multi-view 3D reconstruction across the edge–cloud continuum. We considered a modular pipeline composed of depth estimation, transformer-based multi-view fusion, and point cloud generation with 3D Gaussian Splatting and analyzed its execution characteristics on heterogeneous edge and cloud platforms.

Through experimental evaluation, we demonstrated the strong imbalance between computation and communication costs, primarily caused by large intermediate data generated in early pipeline stages. These results highlight the importance of jointly considering processing and data transfer when determining stage placement. To address this challenge, we proposed an online scheduling approach that dynamically selects execution locations to reduce the cloud cost while satisfying latency constraints through a greedy top-*K* search strategy. By incorporating the system state and network conditions, and by reducing the decision complexity through top-*K* node selection, the proposed scheduler enables efficient and scalable deployment across the edge–cloud continuum. The simulation results show that the proposed approach effectively reduces cloud usage while satisfying latency constraints compared to baseline strategies that execute the entire pipeline on a single node.

As future work, we plan to extend the scheduling model to support the parallel execution of multiple pipeline stages on edge nodes, enabling more efficient resource sharing across concurrent requests. In addition, we aim to deploy and evaluate the proposed scheduling approach on real multi-node edge infrastructure, allowing for a more comprehensive assessment under realistic network conditions and system dynamics. 

## Figures and Tables

**Table 1:** Latency breakdown (seconds) for the four-image pipeline.

Config.	Depth	Tx D→M	Fusion	Tx M→S	Export	Total
E–E–E	1.32	0	1.18	0	3.33	5.91
E–E–C	1.33	0	1.19	0.74	2.13	5.68
E–C–C	1.33	2.55	0.83	0	2.13	7.18
C–C–C	0.55	0	0.81	0	2.14	3.71

## Data Availability

The original data presented in the study, including the simulation results used for the evaluation, are openly available on GitHub at https://github.com/cilicivan/3d-rec-scheduler/tree/main/results (accessed on 2 July 2026).
